# Real-Time Strap Pressure Sensor System for Powered Exoskeletons

**DOI:** 10.3390/s150204550

**Published:** 2015-02-16

**Authors:** Jesús Tamez-Duque, Rebeca Cobian-Ugalde, Atilla Kilicarslan, Anusha Venkatakrishnan, Rogelio Soto, Jose Luis Contreras-Vidal

**Affiliations:** 1 National Robotics Laboratory, School of Engineering and Sciences, Tecnológico de Monterrey, Monterrey N.L. 64849, Mexico; E-Mails: chuy_611@hotmail.com (J.T.-D.); cobian.rivka@gmail.com (R.C.-U.); rsoto@itesm.mx (R.S.); 2 Laboratory for Non-Invasive Brain-Machine Interface Systems, Department of Electrical and Computer Engineering, University of Houston, Houston, TX 77004, USA; E-Mails: a.kilicarslan@gmail.com (A.K.); anushavenkat1@gmail.com (A.V.)

**Keywords:** exoskeleton straps, monitoring system, rehabilitation robotics, spinal cord injury (SCI), pHRi, graphical user interface (GUI), physical therapy, safety

## Abstract

Assistive and rehabilitative powered exoskeletons for spinal cord injury (SCI) and stroke subjects have recently reached the clinic. Proper tension and joint alignment are critical to ensuring safety. Challenges still exist in adjustment and fitting, with most current systems depending on personnel experience for appropriate individual fastening. Paraplegia and tetraplegia patients using these devices have impaired sensation and cannot signal if straps are uncomfortable or painful. Excessive pressure and blood-flow restriction can lead to skin ulcers, necrotic tissue and infections. Tension must be just enough to prevent slipping and maintain posture. Research in pressure dynamics is extensive for wheelchairs and mattresses, but little research has been done on exoskeleton straps. We present a system to monitor pressure exerted by physical human-machine interfaces and provide data about levels of skin/body pressure in fastening straps. The system consists of sensing arrays, signal processing hardware with wireless transmission, and an interactive GUI. For validation, a lower-body powered exoskeleton carrying the full weight of users was used. Experimental trials were conducted with one SCI and one able-bodied subject. The system can help prevent skin injuries related to excessive pressure in mobility-impaired patients using powered exoskeletons, supporting functionality, independence and better overall quality of life.

## 1. Introduction

The development of clinical human-machine systems such as powered robotic exoskeletons and other wearable devices has advanced considerably in the last decade. These robotic devices are now being tested in clinical populations such as spinal cord injury (SCI) and stroke to assess safety, usability, and potential benefits of the technology [1–4]. As these systems require a close physical human-robot interface, it is critical that dynamic and static pressure and shear forces imposed on the patient by the robot are maintained within safe ranges that ensure the health of the skin tissue [5]. Thus, obtaining accurate measurements of forces exerted by strapping systems during physical interactions at the physical interface between the orthosis and the user is essential to ensure safety operation of the device. In the past, researchers have studied the effects of temperature and humidity in human-machine interfaces, specifically in prostheses, in an attempt to characterize comfort and tissue integrity during use [6–9]. Pressure sensors have been developed for static conditions such as sitting in a wheelchair and safety guidelines have been developed for use by wheelchair users [10–13]. Unfortunately, although some research in microclimate sensing in wearable devices is noted in the literature [14], as well as sensor technology for the measure of the physical human-robot interaction pressure [15], little is known about static and dynamic pressure interaction in fastening interfaces found in exoskeletons, in part due to the lack of suitable instrumentation. An exception is the design of a pressure sensor based on mechano-opto-electronic transduction principles reported in [16] that allows for measurement of pressure distributions (or fastening forces) along the length and the width of an exoskeleton's cuff. Initial validation of this system was performed in healthy able-bodied subjects fitted with a powered exoskeleton during treadmill walking. However, pressures during turning and non-locomotor tasks such as standing and sitting were not tested and the system was not evaluated with SCI patients.

The amount of pressure required for necrosis in specific areas is proportional to the amount of muscle and fat tissue present and can rise up to 80 mm·Hg in areas such as thighs [17], whereas areas with skin in close contact with bony structures are most vulnerable to contact pressures, such as the case of skin covering the tibia [18]. The specialized literature sets metrics regarding pressure on the human body at 40–50 mm·Hg for maximum allowance before tissue oxygenation is impaired, and 30–35 mm·Hg for adequate circulation [19]; this is regarded as a safe margin for every part in the body but may vary by 5–10 mm·Hg depending on the specific patient's cardiovascular conditions [20]. The Pressure Pain Threshold (PPT) and Maximum Pressure Tolerance (MPT) were described in a recent study as reaching upwards to 2100–3600 mm·Hg, but such metrics refer to instant pain and lesions [21]. It is known that necrotic tissue is caused by pressures impairing oxygenation of tissue being sustained over time. Such lesions can be prevented by ensuring pressure drops below 40 mm·Hg for a minimum of 10 min with no more than 120 min in between drops. These measures typically allow for proper oxygenation of tissue and prevent the formation of necrotic tissue leading to skin ulcers [22]. Medical knowledge suggests a continuous variation in pressure would prove beneficial to tissue oxygenation by forcing constant circulation of blood along the body (José Contreras Ruiz, Hospital General Manuel Gea González, México City, México; personal communication)

Lesions in cutaneous tissue may also be provoked by friction, sweat-induced humidity and tearing forces caused by external interfaces; such risk factors are typically eliminated by ensuring constant contact with external interfaces and coinciding motion in all interface contact areas as well as the use of materials that allow to breadth or that, additionally, provide the function of moving humidity from the inside of the interface to the outside [18,23].

A large variety of force and pressure monitoring systems are available from developers such as Tekscan (South Boston, MA, USA). These systems are typically focused on research and design applications and operate in pressure ranges much higher than 40 mm·Hg, going up to the thousands in some cases. Development for active assistive application has evidently not been a primary concern. Systems allowing readings of 20–100 mm·Hg and easily integrated to fastening interfaces to provide automatic control for safety are not currently readily available. Thus, the prolonged use of powered orthotic systems is currently challenged by discomfort and potentially dangerous exposure to high pressure levels, as well as other contact phenomena, in the areas of the body where machines are fastened to users [24]. Irritation and injuries leading to necrotic tissue and loss of limbs have, for a long time, been a problem in rehabilitation and all along the lifespan of SCI patients experiencing different levels of mobility impairment: problems are typically presented in areas neighboring the waist due to the use of wheelchairs for mobility and mattresses for rest [25–27]. With the increasing use of powered exoskeletons, similar problems are likely to occur in fastening areas, especially in the lower limbs.

To address the above challenge at the physical interface in human-robot systems, a real-time pressure-monitoring system for fastening systems in powered robotic exoskeletons and other orthotic systems is presented. Validation was conducted in two human subjects, including a patient with paraplegia, using a powered exoskeleton and the proposed pressure monitoring system. Pressure dynamics in the fastening straps of a lower-limb exoskeleton manufactured by Rex Bionics (Auckland, New Zealand) was documented for characterization of pressure levels and patterns with respect to specific locomotive and non-locomotive tasks.

## 2. Materials and Methods

### 2.1. Monitoring System

#### 2.1.1. Hardware

The sensing unit design integrates flexible force sensors wired to a circuit that allows wireless transmission of amplified signals displayed in real time by a Graphical User Interface (GUI) in a personal computer. Tekscan's FlexiForce^®^ Standard Force & Load Sensors Model A401-25 (Tekscan, Inc., Boston, MA, USA) were selected because they allow individual monitoring of small contact areas (0.203 mm), have a suitable dynamic range, and can be arranged to cover the entire physical interface with an amount of sensors that is easily manageable. These sensors allow measurements of force magnitudes in the range of 0–111 N, and pressures as low as 15 mm·Hg. Sensor construction is based on a printed circuit consisting of two conductive strips and the active sensor region, pressed by two transparent polyester film layers. The active region, a circle of 2.54 cm in diameter, varies its electrical resistance inversely to the applied force. Table 1 shows technical specifications for the system. Circuit schematics and additional details can be found in Figures S1 and S2 and Table S1 of the Supplementary Material.

All sensors are installed in pressure-distributing pads constructed from closed-cell nitrile/vinyl blended and resilient foam used in COTS Apache Mills 39-098-0900 knee-saver protection mat (Apache Mills Inc., Calhoun, GA, USA). The pads distribute forces over the contact area, reducing pressures experienced by the user of a given interface. Material was selected to minimize risk factors like friction, sweat-induced humidity and tearing forces caused by external interfaces.

**Figure 1.**
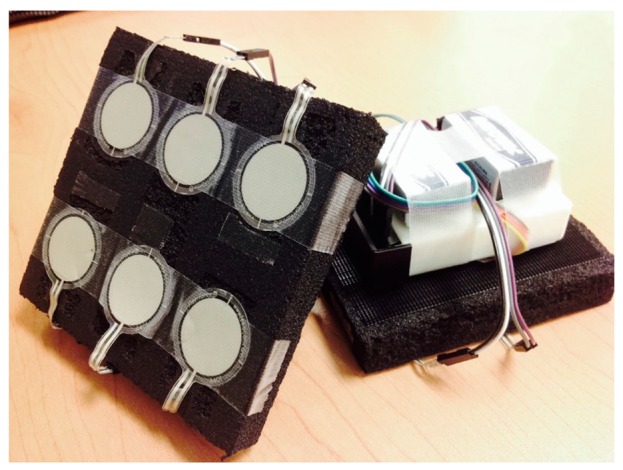
Front and back of one sensor array, showing Tekscan's FlexiForce^®^ Standard Force & Load Sensors Model A401-25. Dimensions: 11.5 × 11 × 2 cm for shins; 12 × 11.5 × 2 cm. for thighs. Approximate weight with batteries: 190 g.

Materials, the number of pads used, and their shapes can vary depending on the application. Specific sensor position can also vary. In the case of the Rehab Rex lower-limb exoskeleton, four pads were used in total—one on each fastening interface. Interfaces securing the users' thighs were fit with pads including six sensors each, as shown in Figure 1. Interfaces securing the shins contained a matrix with four sensors. The circuit integrates a 6-channel amplifier, designed and constructed based on the recommended drive circuitry for Flexiforce^®^ sensors [28]. Amplified signals were connected to an 8-channel CMOS analog multiplexer in conjunction with a Wixel^©^ (Pololu Corp., Las Vegas, NV, USA) to order data input from the sensing unit and perform wireless communication.

The circuit was assembled using a custom printed circuit board and surface mount electronics to minimize size and weight. The system supports up to 6 sensors per sensing unit. A power source of 5 V is required to power the Wixel^©^ and the Op-amps, −5V is used to power the sensors. For data transmission, each multiplexor output is connected into a different input in the wireless module to facilitate software processing and due to bit-restriction in data packets. The Pololu Wixel^©^ is a general-purpose programmable module featuring a 2.4 GHz radio and USB, its operation is based on the CC2511F32 microcontroller from Texas Instruments^®^ (Dallas, TX, USA), this device was selected to transmit the data because of its compactness and ease of use. The paired, receiving Wixel^©^ is powered by the USB cable, which is connected to the processing PC. Transmission was achieved with a 0.02 s delay. Currently, the system is operational, following Tekscan Flexiforce Sensor's technical recommendations, for applications requiring measuring of pressures <3.33 Hz. The system, however, can measure pressures of durations as short as 75 ms (with proper calibration), as shown by the dynamic-load tests reported below, and can also easily be modified to fit sensors with higher bandwidths.

#### 2.1.2. Software

The software was designed to record and display real time measurements through a GUI, it was developed in Matlab (The MathWorks, Inc., Natick, MA, USA) but stands alone as an independent executable application. The GUI allows one to select recording and viewing preferences. Viewing options include a heat map configuration where each pad is shown independently, showing information in mm·Hg according to sensor calibration and adjusted to visually decay from each sensor's position center to approximate real behavior of pressure distribution in human tissue. Bar and line graphs that describe each individual sensor are also available.

For faster data logging, visual output may be disabled and replaced by a timer that automatically timestamps all readings recorded. An option to run calibration before each use is displayed in case it is required. Calibration files may be recorded for later use. The system integrates UDP communications to include relevant task-oriented instructions in the data logs, when communication of such data is available in the interface system. This is especially beneficial when attempting characterization of tasks. Data interpretation is performed by receiving individual packets, each corresponding to a complete pad and sensor array.

The Wixel^©^ unit was programmed to label each input accordingly before transmission and the software acknowledges such tags for proper sorting. Alerts for excessive pressure and additional assistive and user-oriented features have been included for practical applications. For the application with Rehab Rex lower-limb exoskeleton, the heat map visualization has been customized to accommodate the sensor layout used with this exoskeleton's interface.

#### 2.1.3. Calibration

For each calibration trial, two sets of measurements were performed. Each set required placing four calibrated weights with different masses, one by one, over each of the 20 sensors for a total of 80 readings per run. This process allowed for an individual description of units output by the system, for each sensor, with regards to the pressure input. 200 g, 300 g, 500 gr and 1 kg were used as the four weights tested (Ajax Scientific, Scarborough, ON, Canada). Contact with the entire sensing area (5.066 cm^2^) was ensured during 15 s to allow for a stable output value. The maximum stable-state output value for each sensor, considering both runs, was related to the corresponding pressure input in mm Hg. A linear regression was estimated from points recorded to describe the transfer function of the measuring system for each sensor. The average value for R^2^ was 0.9766.

#### 2.1.4. Dynamic Load Testing

**Figure 2.**
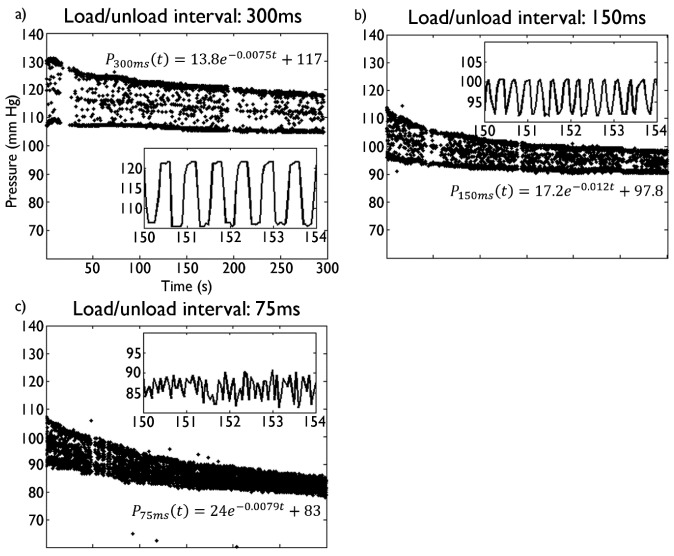
Dynamic load trials with load/unload pressures of 132 and 110 mm Hg. (**a**) 300 ms load/unload intervals; (**b**) 150 ms intervals; (**c**) 75 ms intervals. Note the exponential decrease in the pressure measurements as a function of time, particularly for intervals <300 ms, which would require sensor calibration. Insets show load/unload behavior. The exponential decay fit for the maximum pressure measured is also shown.

In these tests, a load of 130 mm·Hg was applied for intervals of 300, 150 and 75 ms, demonstrating noticeable changes in sensor pressure during prolonged exposure to intermittent loads at frequencies of 3–16 Hz (Figure 2). Pressure readings were underestimated for pressure loads applied for brief periods of 150 and 75 ms. Thus, the proposed sensor bandwidth is estimated to be ∼3 Hz, which is sufficient given the slow speed gaits expected with SCI patients wearing an exoskeleton (pressure stimuli with durations of under 300 ms are unlikely and this does not pose a significant problem). Bending tests were performed with radii of up to 36.4 mm, resulting in consistent increases in pressure measurements of up to 16.92 mm Hg. Detailed results are shown in supplementary Figure S3.

#### 2.1.5. Cushioning Effect

**Figure 3.**
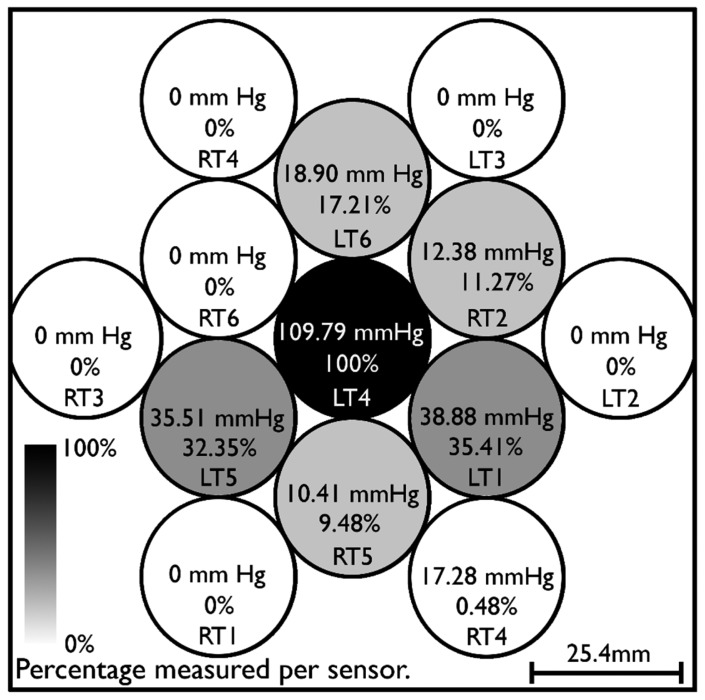
Distribution of pressure over 13 sensors due to cushioning pad. Pressure of 445.3 mm·Hg was applied directly over LT4 sensor (5.067 cm^2^) in the center.

Pressure is distributed by the cushioning pad used in the design. This means, as seen in Figure 3, that an average of 10%–35% of pressures applied to given spatial location on the interface will be measured by sensors in the vicinity.

This suggests that pressure variation within a single sensor could not varied by a significant factor as a misreading of a safe level compared to a pain level within a sensing area would only happen if a 97% difference (e.g., 60 mm Hg compared to 2000 mm·Hg) were to take place within the 5 cm^2^ area. Tests performed with different weights (1 kg, 3 kg, 5.5 kg) indicate the cushioning material distributes force in a way such that the area directly under pressure withstood only around 25% of the actual pressure and the whole area of the interface, which is covered by the cushioning pad, withstood approximately 50% of the applied pressure due to the material's distribution capacity (which technically increases the area where the same force is being applied) regardless of the pressure being applied.

### 2.2. Methods

Experimental Protocol

Testing of the device was performed in adult human subjects after obtaining informed consent approved by the Committee for the Protection of Human Subjects (CPHS) at the University of Houston, where experiments were carried out. One able-bodied subject and one SCI patient performed the following dynamic-condition tasks, in sequence, for data collection: sit to stand, walk forward, turn 180° to the right, turn 180° to the left, and stand to sit. Real time force measurements were logged using the system's computer application. Visualization of data was disabled during logging to achieve 20 Hz readings. All start and stop signals corresponding to the performed tasks were logged simultaneous to force measurements in an SD card. Kinematics data could afterwards be synchronized with pressure data. All data was logged with its corresponding time stamp. Experimental trials performed by the SCI patient were also recorded in video. All procedures regarding exoskeleton fitting were performed manually as customary, to provide data about dynamics encountered in regular conditions. Physical therapists adjusted, manually and by visual inspection, tightness according to experience and protocol. All tasks were performed at 100% speed of Rehab Rex's default settings, which translates into forward walking speeds of 0.3 km/h.

## 3. Results

**Figure 4.**
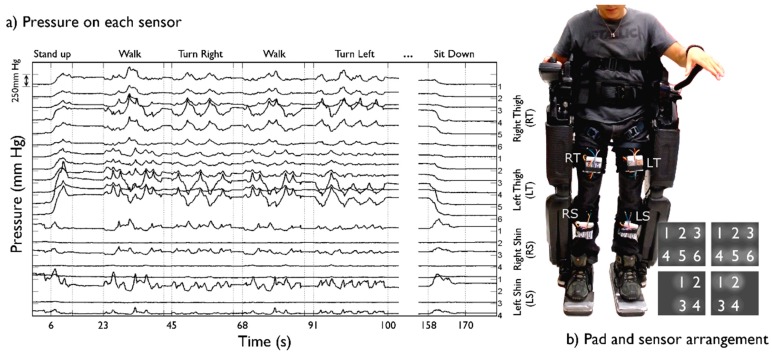
Pressure measurements of one SCI subject performing locomotion and non-locomotion tasks using a powered exoskeleton. (**a**) Twenty sensors arranged in 4 measuring pads (RT, RS, LT, LS). One trial including “stand up”, “walk”, “turn right”, “turn left” and “sit down” tasks. LT sensors 5 and 6 consistently show greater pressure levels, suggesting the lower strap of the subject's left thigh was adjusted tighter with the outer part receiving more force; (**b**) Rehab Rex exoskeleton from Rex Bionics is shown. Six sensors are used on thigh pads; 4 on shin pads. Shin sensors are positioned over risk areas caused by bony prominences corresponding to the tibia.

**Figure 5.**
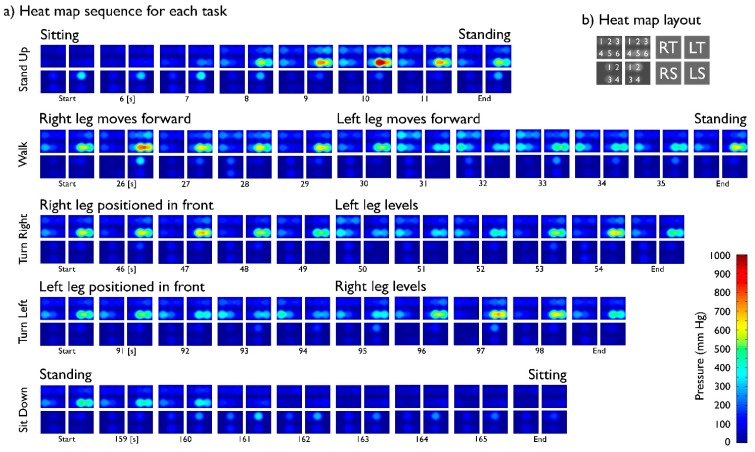
(**a**) Each set of four heat maps corresponds to pressure distribution on both legs for each indicated robot motion during a typical testing protocol. Data corresponds to that shown on Figure 4. Greater tension in the lower strap adjusting the subject's Left Thigh is evident. Pressure is consistently greater in subject's thighs, suggesting straps on the upper leg support most of the exoskeleton's user's weight. Pressure increases on Shins in sitting position. As the trial advances, base pressure on the legs appears to drop: compare “End” heat maps for the first four tasks, all corresponding to standing position; (**b**) Diagram used for heat map interpretation. RT: Right Thigh; LT: Left Thigh; RS: Right Shin; LS: Left Shin.

Pressure dynamics in the fastening straps from the Rehab Rex lower-limb exoskeleton were successfully recorded for both subjects. Figure 4 depicts data from a complete protocol run for the SCI patient, whereas Figure 5 shows heat maps corresponding to selected segments of the same run. Greater relative pressures in the lower part of the left thigh are evident, especially towards the outer part of the leg. This is likely consequence of uneven tensioning of the straps during the standard fastening procedure.

Pressure shifts in between legs are also evident during the walking tasks, with the supporting leg experiencing an increase in pressure whereas a decrease is observed in the swinging leg. Because of the uneven tensioning of straps leading to a greater relative pressure on the left thigh, pressure is constantly visible (see Figure 5a); nevertheless, the “walk” and “turn” tasks evidently produce a drop in skin pressure.

Pressures were unexpectedly low on both shin interfaces while performing tasks in an upright position, e.g., during walking or turning. A decrease in pressure was measured in both straps fastening the shins as the subject performed the “stand up” task and a coinciding increase was measured when the “sit down” task was performed at the end of the run. Pressures in both thigh interfaces were lower when sitting. Pressures remained focused on the thigh straps in the Rehab Rex lower-limb exoskeleton during upright performance.

**Figure 6.**
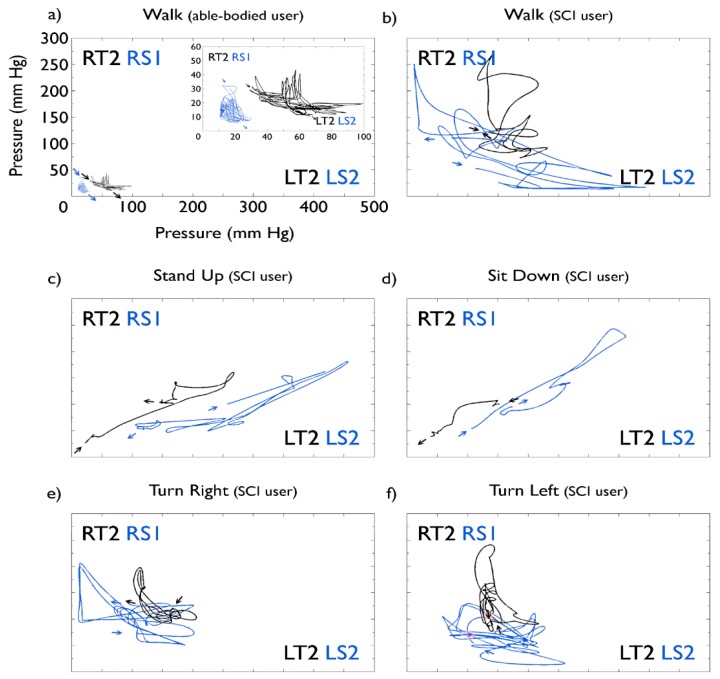
Pressure measured on Right Thigh compared to Left Thigh (black) and Right Shin compared to Left Shin (blue). Sensor pairs with representative interactions were used. Arrows signal starting and ending points for each task. All portraits use the same scale. Data corresponds to that shown in Figures 4 and 5. (**a**) “walk” task performed by an able-bodied subject, used as control reference for performance by SCI subject. The inset shows in more detail the pressure dynamic patterns (note the different scales). 8 steps forward were performed; (**b**) “walk” task by SCI subject: three steps forward were performed; (**c**) SCI subject: sitting to standing position, once; (**d**) SCI subject: standing to sitting position, one trial. (**e**,**f**) SCI subject performed a 180° turn toward his right and left, correspondingly, taking 3 partial-turn steps of 60°, each, to complete the turn.

Phase portraits depicted in Figure 6 show a comparison of pressures measured across the legs, showing pressures in the thighs in black and pressures in the shins in blue. Panels (c) and (d) clearly show the inverse pressure dynamics of “stand up” and “sit down” task, with the ending pressure point of the former being the starting pressure point of the latter in both the thigh and the shin. Pressures evidently remained consistent towards the end of the run compared to the start, despite the constant shifts through the protocol.

Panels (a) and (b) correspond to “walk” tasks for the able-bodied control subject and the SCI patient, respectively. Comparison shows interface pressures to be much greater when the exoskeleton is used by the SCI patient, compared with the able-bodied subject. Maximum averaged pressure experienced by the able-bodied subject on thighs was 140 (right) and 220 (left) mm Hg and on shins 25 (right) and 40 (left) mm Hg. Maximum averages for the SCI patient were 208 (right) and 282 (left) mm Hg on the thigh interfaces and 82 (right) and 115 (left) mm Hg on the shins.

Figure 6a also shows a zoom of the “walk” pressure dynamics on the able-bodied control subject. A clear pattern is shown for pressures on the thighs, with a negative correlation between pressures on each leg. Even though pressures on the shins are low, at around 30 mm·Hg, the same pattern as with the thighs can still be identified.

## 4. Discussion

A novel real-time pressure measuring system for fastening straps in physical human-orthotic systems has been developed and validated in both able-bodied and SCI subjects. Data acquired with the proposed measuring system, including hardware and software, successfully validates its functionality, pressure ranges, and usability across various locomotion and non-locomotion tasks. The wireless transmission, refresh rate, pressure resolution, pressure range, and software connectivity proved to be sufficient for integration with human-machine interface for real-time alerts of pre-determined pressure thresholds. Data acquisition and processing also proved useful for integration with control systems for strap-tension variation.

With pressures reaching 1000 mm·Hg in two thigh sensors, attention must be paid to the usage protocols specified for exoskeleton use. Oxygenation-impairing pressures were observed at over 40 mm·Hg, along with pressure pain thresholds (PPT) at around 2100 to 3600 mm Hg [21] in the thigh area, which in this case contacts the fastening interface. Surpassing the first threshold is expected and does not in any way pose a threat to the user's health; surpassing the second threshold could pose a problem to the user if sustained for long periods of time.

Pressures oscillated in the lower part of the left thigh during the first 30 s of the task and then dropped significantly when “walk” tasks were performed. All other sensor pressures remained below PPT for the entire trial. Minimal adjustments in strap tension would ensure avoidance of greater pressures in specific sensors and a real-time display would allow for physical therapists to make such adjustments accurately. Ensuring tasks such as “walk”, which shift pressure from one interface to another and allow for variations of pressure, would minimize the risk of injuries related to excess pressure during exoskeleton usage. While performing tasks upright, pressures dropped intermittently to under 40 mm·Hg for all sensors on the shins but not so for sensors on the thighs, with most sensor readings dropping to around 80–120 mm·Hg, when lowest. To ensure proper oxygenation of the tissues, resting periods in sitting position, with a duration of at least 10 min, should be implemented into usage protocols. Shin pressure would likely increases in this position but will decrease when upright.

Maintaining the same strapping protocol, pressures measured for the able-bodied subject used as control were significantly lower than those measured for the SCI patient, in both thighs and shins; this could be attributed to the fact that SCI patients have no support from their lower body and their full weight is being carried by the strapping mechanisms in the exoskeleton, while an able-bodied subject can hardly withhold himself from using his legs for support.

Our system can be compared to the pressure sensor described in [16], which has also been developed for exoskeleton applications. In [16], the sensor was based on a mechano-opto-electronic principle whereas our system is based on low cost, COTS FSR sensors customized into a sensor array. Importantly, our system represents a complete wireless, modular, stand-alone system, validated in able-bodied and SCI subjects performing both locomotive (walking, turning) and non-locomotive tasks such as sitting and standing. A system to measure pressure in feet during standing has been presented in [29]. The authors reported the potential for peak-pressure underestimation in relation to sensor dimensions and suggested an optimal sensor size of 100 mm^2^ for relevant applications with flat, hard, interfaces. Our own tests, conducted with cushioning material for use in fastening interfaces, supports the conclusions in [29] which indicate larger sensor dimensions can be used when coupled with soft pressure-distributing interfaces.

## 5. Conclusions

Patients with compromised immune systems, friable skin or with limited sensory, including pain, perception, need special monitoring and care when fitted to exoskeletons and other wearable human-machine systems. Elderly and SCI patients with underlying medical conditions are susceptible to skin tears and pressure sores, which can be life threatening. The proposed pressure monitoring system described herein, can be used to monitor and characterize static and dynamic pressure in physical interfaces during locomotive and non-locomotive tasks and use this information for risk mitigation purposes. The proposed system could be deployed in various applications ranging from seatbelts to straps and braces in rehabilitation robotics and other physical human-machine interfaces.

## Supplementary Materials

Supplementary materials can be accessed at: http://www.mdpi.com/1424-8220/15/2/4550/s1.



## Figures and Tables

**Table 1.** Technical specifications of pressure sensor system.

**Component**	**Specification**	**Details**
**Wireless data transmission**	Module	Wixel USB module
	Sampling rate	20 Hz, 0.02 s delay
	Protocol	Serial

**Force sensors**	Force range	0–25 lb (111 N)
	Bandwidth	3.33 Hz
	Sensing Area	5.067 cm^2^, 25.4 mm (91 in.) diameter per sensor. 30.4 cm^2^ with 6 sensors. Different sensors can be used to vary area covered.
	Thickness	0.203 mm (0.008 in.)
	Length	56.9 mm (2.24 in.)
	Width	31.8 mm (1.25 in.)

**PCB**	Operating voltage	5 V
	Operating current	43 mA D.C.
	Number of sensors	Up to 6 sensors per array
