# Supplementary material for: Real-Time Strap Pressure Sensor System for Powered Exoskeletons
Source: Sensors (Basel). 2015 Feb 16;15(2):4550–63. doi: 10.3390/s150204550 (PMC4367424; doi:10.3390/s150204550)
Supplement: Supplementary file 1 [file sensors-15-04550-s001.pdf]

OPEN ACCESS

sensors

ISSN 1424-8220

www.mdpi.com/journal/sensors

Supplementary Information

Real-Time Strap Pressure Sensor System for Powered Exoskeletons. *Sensors* 2015, *15*, 4550-4563

Jesús Tamez-Duque [1], Rebeca Cobian-Ugalde [1], Atilla Kilicarslan [2], Anusha Venkatakrishnan [2], Rogelio Soto [1] and Jose Luis Contreras-Vidal [2,]*

[1] National Robotics Laboratory, School of Engineering and Sciences, Tecnológico de Monterrey, Monterrey N.L. 64849, Mexico; E-Mails: chuy_611@hotmail.com (J.T.-D.); cobian.rivka@gmail.com (R.C.-U.); rsoto@itesm.mx (R.S.)

[2] Laboratory for Non-Invasive Brain-Machine Interface Systems, Department of Electrical and Computer Engineering, University of Houston, Houston, TX 77004, USA; E-Mails: a.kilicarslan@gmail.com (A.K.); anushavenkat1@gmail.com (A.V.)

* Author to whom correspondence should be addressed; E-Mails: jlcontreras-vidal@uh.edu; Tel.: +1-713-743-4429.

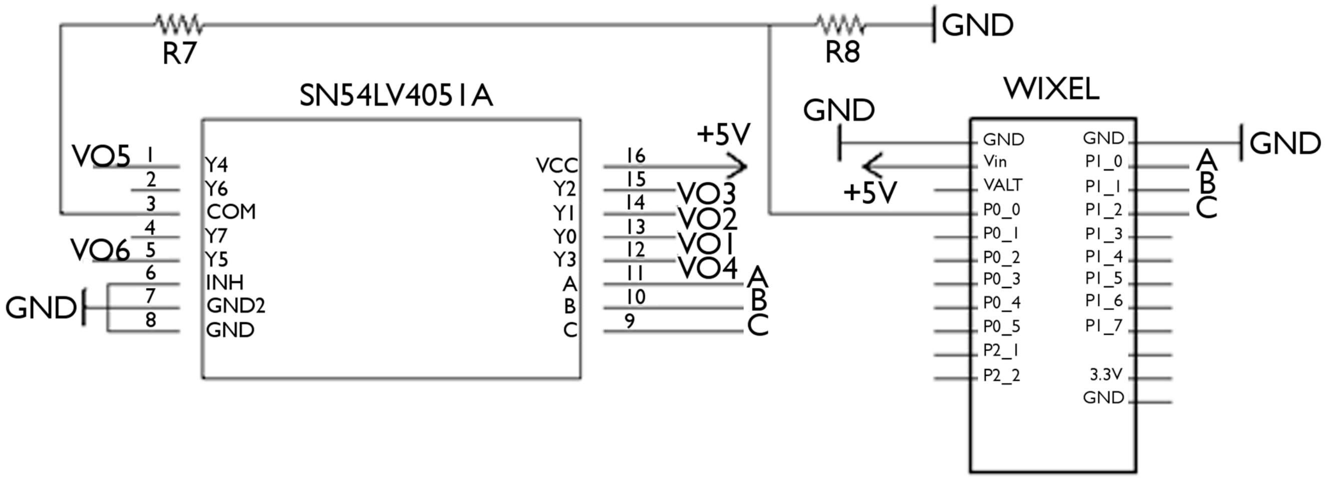

Figure S1. Multiplexor with sensor inputs VO1-VO6. Wixel module is included for wireless transmission of data.

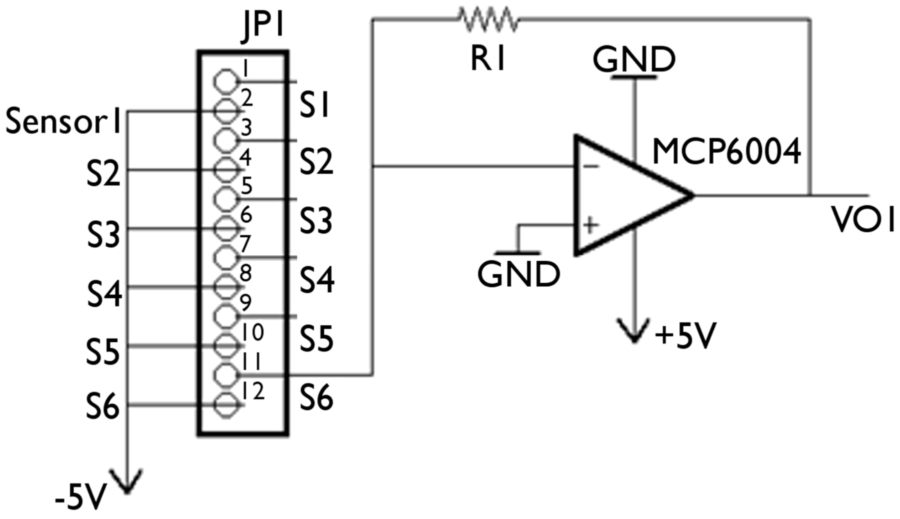

Figure S2. Electronic configuration used for each sensor.

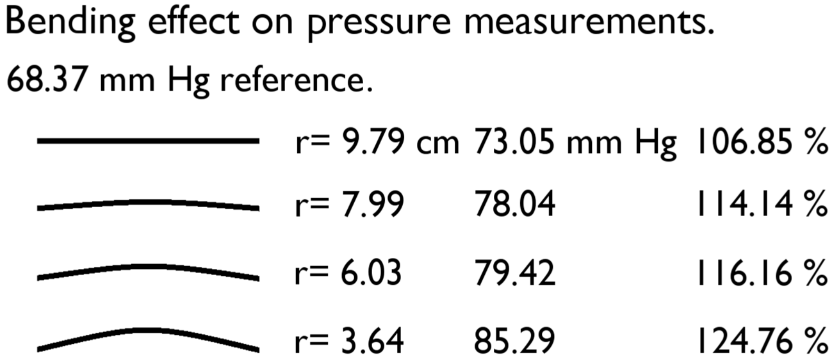

Figure S3. Measurement variations with respect to sensor bending.

Table S1. Component details for the developed circuit shown in Figures 6 and 7.

Name on Schematic	Component
S1–S6	Tekscan's FlexiForce® A401-25 Force Sensors
SN54LV4051A	CMOS Analog Multiplexer
WIXEL	Polulu Wixel©
MCP6004	Op-amp
R1-R6	20k Resistor (1% tolerance)
R8	2.2k Resistor (1% tolerance)
R7	1.8k Resistor (5% tolerance)

© 2015 by the authors; licensee MDPI, Basel, Switzerland. This article is an open access article distributed under the terms and conditions of the Creative Commons Attribution license (http://creativecommons.org/licenses/by/4.0/).
